# Sociodemographic and behavioral factors associated with time to first cigarette upon waking: Findings from the Malaysian national tobacco survey 2023

**DOI:** 10.18332/tid/218817

**Published:** 2026-05-22

**Authors:** Pei Pei Heng, Thamil Arasu Saminathan, Tania Gayle Robert Lourdes, Jia Hui Lim, Muhammad Fadhli Mohd Yusoff

**Affiliations:** 1 Institute for Public Health, National Institutes of Health, Ministry of Health, Shah Alam, Malaysia; 2 School of Pharmacy, Faculty of Health and Medical Sciences, Taylor’s University, Subang Jaya, Malaysia

**Keywords:** time to first cigarette, behavioral factors, GATS-M, TTFC

## Abstract

**INTRODUCTION:**

Time to first cigarette (TTFC) upon waking has been recognized as a robust behavioral marker for assessing the severity of nicotine dependence. Given that dependence is a primary driver of sustained tobacco use, which significantly impedes successful cessation efforts, this study aimed to determine the prevalence and associated determinants of TTFC within 30 minutes upon waking, among current smokers in Malaysia.

**METHODS:**

Data were derived from the Global Adults Tobacco Survey Malaysia (GATS-M) 2023, a cross-sectional study employing multi-stage proportionate-to-size sampling to recruit 4269 represent sample of non-institutionalized populations in Malaysia aged ≥15 years. The dependent variable was ‘Time to first cigarette’ (TTFC) upon waking up (within or more than 30 minutes); while independent determinants investigated including sociodemographic variables (gender, ethnicity, education level and marital status), and behavioral clues: age of smoking initiation, previous quit attempt, future quit intention, perceived difficulty in quitting, and apprehension about adverse health outcome. Data were analyzed using SPSS version 25, descriptively and with multivariable logistic regression to determine the associated factors of TTFC within 30 minutes of waking up.

**RESULTS:**

Approximately 29.2% among the current smokers reported taking the first puff of smoke within 30 minutes upon waking. Multivariable logistic regression substantiated that smoker who initiated smoking before the age of 18 years (AOR=1.60; 95% CI: 1.09–2.76), those without previous quit attempt (AOR=1.59; 95% CI: 1.39–2.01), smokers perceived difficult to quit (AOR=1.48; 95% CI: 1.08–2.54), and those who were not worried about the adverse health outcome of smoking (AOR=2.04; 95% CI: 1.17–3.54) were significantly more susceptible towards TTFC within 30 minutes of waking up.

**CONCLUSIONS:**

This finding suggests that highly dependent smokers exhibiting these behavioral characteristics may benefit from targeted behavior-based interventions to address nicotine dependence.

## INTRODUCTION

Although age-standardized global smoking prevalence has declined over recent decades, substantial regional disparities persist, and the absolute burden of tobacco-attributable disease remains high in low- and middle-income countries, particularly in Southeast Asia^1^. Forecasts predict the increment in the absolute number of global cardiovascular (CVD) deaths attributable to tobacco to approximately 3.267 million by 2045, and disability-adjusted life-years (DALYs) lost annually are projected to potentially increase to 75.755 million by 2045^2^. Smoking has a devastating impact on Malaysia, causing a significant number of premature deaths and disabilities, an estimated 20000 Malaysians die each year due to smoking-related illnesses^2^. On the other hand, the economic cost is substantial, with approximately MYR 2.9 billion (1000 Malaysian Ringgit about US$250) or 16.5% of the national health expenditure, allocated to treat cardiopulmonary diseases and lung cancer that are directly linked to tobacco use^3^. Despite two decades of implementing various anti-tobacco policies consistent with the WHO Framework Convention on Tobacco Control (FCTC) which include smoke-free regulations, pricing strategies, advertising restrictions, and packaging mandates, the prevalence of tobacco use in Malaysian adults has remained stagnant at around one-fifth^4^. While FCTC-endorsed measures are well-established as effective when comprehensively implemented and rigorously enforced, variations in implementation fidelity, enforcement capacity, sociocultural norms, and ongoing tobacco industry interference may influence their population-level impact in Malaysia. In addition to these structural considerations, the highly addictive nature of nicotine remains a critical factor sustaining tobacco use and impeding successful cessation.

This persistent challenge is largely attributed to the highly addictive nature of nicotine, leading to significant dependence among users, which subsequently impedes successful cessation efforts^5^. Studies in Malaysia have consistently reported low sustained quit rates, often attributed to strong nicotine withdrawal symptoms, persistent socio-behavioral influences, and continued exposure to environmental smoking cues^5,6^. The persistence of such cues may reflect gaps in the enforcement of smoke-free regulations, contextual sociocultural acceptance of smoking in certain settings, and adaptive marketing strategies by the tobacco industry. These interrelated factors collectively contribute to cycles of dependence and relapse, thereby attenuating the full potential impact of broader public health interventions. Nicotine dependence is a primary driver of sustained tobacco use and a significant barrier to successful cessation globally, and Malaysia is no exception. The pharmacological effects of nicotine lead to a strong physical and psychological dependence, shaping an individual’s smoking behavior into a compulsive habit often resistant to change. This dependence manifests as intense cravings, withdrawal symptoms including irritability, anxiety, difficulty concentrating, and depressed mood, which in turn manifests upon cessation attempts^7^.

A number of published tobacco-related studies have recognized ‘time to first cigarette upon waking up’ (TTFC) as a robust and widely used behavioral marker for assessing the severity of nicotine dependence^8^. This single-item measure is included in the instrument of the Fagerström test for nicotine dependence (FTND), which is indicative of physiological dependence due to its direct correlation with overnight nicotine withdrawal and the subsequent urgency to alleviate these symptoms^8^. A shorter TTFC, such as smoking the first cigarette 30 minutes of waking consistently, predicts higher levels of nicotine metabolites, a greater exposure to carcinogens, and increased difficulty in achieving and maintaining long-term abstinence. Furthermore, individuals with a shorter TTFC often exhibit more severe withdrawal symptoms and are at a higher risk of relapse during cessation attempts, irrespective of the total number of cigarettes smoked daily^9^. Taken together, this evidence supports the use of TTFC as an important clinical and research indicator for identifying highly dependent smokers and informing cessation strategies.

The smokers’ profile, tobacco consumption habits, patterns, and behavior are among the well-recognized determinants of nicotine dependence^10^. Therefore, understanding and addressing the multifaceted nature of nicotine dependence, encompassing its behavioral, physiological, and contextual dimensions, is critical to improving smoking cessation outcomes and reducing the persistently stagnant prevalence of tobacco use among Malaysian adults^10^. Nevertheless, because of variations in sociocultural background, norms, population characteristics, national laws, and control measures, these predictors may be region-specific^3^. As such, findings from other countries may not be directly generalizable to the Malaysian context. Therefore, a thorough investigation of the determinants of nicotine dependence, as measured by TTFC, within the Malaysian community is imperative. Understanding how individual-level dependence interacts with broader structural and contextual factors may provide complementary evidence to strengthen implementation strategies, enhance targeted cessation support, and optimize the impact of existing national tobacco control policies.

Research on TTFC has been extensively conducted across numerous countries worldwide, especially in high-income regions like United States^9^, Europe^11^, and China^12^. However, most of these studies have emphasized its clinical and biological implications, with comparatively limited attention to its application as a public health stratification tool. A notable gap remains in its comprehensive examination from a community or public health perspective. Although smokers with high nicotine dependence often demonstrate lower cessation success, identifying individuals with TTFC ≤30 minutes provides actionable information beyond prognosis alone. TTFC-based stratification may guide the intensity, modality, and allocation of cessation support. For instance, smokers with shorter TTFC may benefit from early initiation of combination nicotine replacement therapy, higher pharmacotherapy dosing, extended treatment duration, proactive follow-up, and more intensive behavioral counselling. At the population level, TTFC can help identify communities with a high concentration of heavily dependent smokers who may require resource prioritization, targeted outreach, or specialized cessation services.

In Malaysia, most local studies have been small-scale surveys focusing on factors associated with smoking initiation, with limited exploration of nicotine dependence severity at the population level. Individual-level associations, therefore, remain insufficiently established, particularly regarding how TTFC prevalence and its associated factors vary across Malaysia’s diverse sociocultural settings. Moreover, the potential utility of TTFC as a simple, cost-efficient screening indicator within primary care and community health programs remains underexplored. Understanding the sociodemographic and behavioral determinants of TTFC ≤30 minutes could inform differentiated cessation pathways, enhance risk stratification at primary care, optimize resource allocation, and strengthen tobacco control strategies tailored to the Malaysian context. This study aimed to determine the prevalence and associated sociodemographic and behavioral factors of TTFC within 30 minutes upon waking up, among Malaysian smokers.

## METHODS

### Study design and sampling

Data were derived from the nationwide cross-sectional study of the Global Adult Tobacco Survey, Malaysia (GATS-M) 2023. GATS-M 2023 is a nationally representative household survey; all households selected through the multi-stage proportionate-to-size sampling procedure were approached for participation. Eligible respondents were non-institutionalized Malaysian residents aged ≥15 years. Individuals residing in institutional settings like prisons, hospitals, military barracks, and hostels were excluded from the sampling frame. In the first sampling stage, the primary sampling units of enumeration blocks (EBs), which were artificial geographical areas created by the Department of Statistics Malaysia, were selected by stratified sampling. Subsequently, the secondary sampling units of living quarters (LQs) were selected via simple random sampling. The detailed methodology of GATS-M was published previously^13^. GATS-M 2023 randomly sampled 5780 households. From each participating household, one eligible individual was randomly chosen to complete the survey. A total of 4269 respondents completed the questionnaire, with an overall response rate of 81.5%. The ethical clearance was obtained from the Medical Research and Ethical Committee, Ministry of Health Malaysia (Approval ID NMRR 20-240-52856).

### Instrument and measure

The GATS-M study utilized a standardized and structured questionnaire developed by the Centre for Disease Control and Prevention and the World Health Organization (WHO). The tool has been translated into the local native language of Bahasa Melayu by a board of content and language experts, pre-tested, and validated. Relevant information was obtained from the selected respondents via a face-to-face approach, using handheld computer devices by trained research assistants. The objective and scope were explained in detail; written consent was obtained before commencing the interviews.

Only current smokers were included in the analysis. A current smoker is defined based on an item of ‘currently smoke tobacco on a daily basis or less than daily’, with those who answered ‘not at all’ being excluded. The dependent variable of ‘Time to first cigarette’ (TTFC) upon waking up was assessed using the question: ‘How soon after you wake up do you usually have your first smoke? Would you say within 5 minutes, 6–30 minutes, 31–60 minutes, or more than 60 minutes?’. Responses were dichotomized into two categories: within 30 minutes and more than 30 minutes, as smoking within 30 minutes of waking is widely considered a strong indicator of nicotine dependence^9^. Independent variables included sociodemographic characteristics such as gender (male, female), ethnicity (Malay, Chinese, Indian, Other), education level (primary or lower, secondary, tertiary), and marital status (single, married). Smoking-related behavioral characteristics and perceptions were also examined, including: age of smoking initiation (<18 years, ≥18 years); quit attempt in past 12 months (yes, no); quit intention in next 12 months (yes, no). The quit attempt and quit intention items were derived directly from the standardized WHO Global Adult Tobacco Survey (GATS) questionnaire, which aligns with MPOWER indicators; no additional researcher-defined criteria were applied. Smoking cessation behavior was assessed by asking: ‘How easy or hard would it be for you to quit smoking if you wanted to?’. Those who answered ‘very easy’, ‘somewhat easy’ were categorized as ‘not difficult’, while those who selected ‘somewhat hard’ and ‘very hard’ were classified as ‘difficult’. In addition, apprehension about adverse health outcome was defined by an item: ‘How worried are you that smoking will damage your health in the future?’. Participants who responded ‘not at all worried’ were categorized as ‘not worried’, whereas those who selected ‘a little worried’, ‘moderately worried’, or ‘very worried’ were categorized as ‘worried’. Chronological age was not included as a separate covariate, as age of smoking initiation was incorporated to reflect developmental exposure and smoking trajectory.

### Data management and analysis

Data were cleaned and weighted according to study design, non-response rate, and corrected for age groups, sex, and urban-rural locality. All analyses were performed with SPSS version 25 statistical software, and results were presented in 95% confidence interval (CI) with p<0.05 being statistically significant. The sociodemographic attributes were illustrated descriptively in frequency (n) and percentage (%). Pearson chi-squared analysis was employed to describe the association between TTFC and all categorical independent variables. All independent variables with p≤0.25 in univariate analysis were included in the model of multivariable logistic regression to determine the associated factors of TTFC. This screening criterion using the cutoff value of 0.25 follows the ‘Purposeful Selection of Covariates’ strategy, which aims to retain not only statistically significant variables but also potential confounders that may not demonstrate a strong association in univariable analysis but could become important in the multivariable model. This structured model-building approach ensures model stability and reduces residual confounding^14^. Two-way interactions were examined among the variables included in the final model: ethnicity, age of smoking initiation, quit attempt, quit intention, perceived difficulty of quitting, and apprehension about adverse health outcomes, in order to assess whether the association between each variable and TTFC differed by the level of another variable. Adjusted odds ratios (AORs) were estimated, and a p<0.05 was considered statistically significant.

## RESULTS

### Sample characteristics and prevalence of TTFC

Only respondents who self-reported themselves as current cigarette smokers were included in the analysis. A total of 686 (16.1%) among the total respondents of 4269 were current smokers. More than one-fourth of current smokers (29.2%) reported taking the first puff of smoke within 30 minutes upon waking, while 70.8% declared TTFC more than 30 minutes. Descriptively, the proportion of current smokers was significantly higher among the male respondents (96.9%), the Malay descent (60.6%), the secondary education attainer (55.3%), the married respondents (68.0%), and smokers who initiated smoking at the age of ≥18 years (53.7%). Scoping into the smoking behavior and perception, the majority of current smokers admitted without a quit attempt in the past 12 months (53.8%) and no quit intention in the next 12 months (80.5%). Additionally, a huge proportion perceived difficulty in quitting (66.4%), and were worried about the adverse health outcome following smoking (56.1%) (Table 1).

**Table 1:** Descriptive sociodemographic and behavioral characteristic of current smokers aged ≥15 years in Malaysia, Global Adult Tobacco Survey-Malaysia (GATS-M) 2023 (N=686)

*Variable*	Estimated population*	*Sample n*	*Percentage %*	*95 % CI*
**Time to first cigarette** (minutes)				
≤30	1049538	227	29.2	24.7–34.1
>30	2550355	459	70.8	65.9–75.3
**Gender**				
Male	3487365	667	96.9	94.0–98.4
Female	112529	19	3.1	1.6–6.0
**Ethnicity**				
Malay	2183178	399	60.6	54.2–66.7
Chinese	384389	54	10.7	7.2–15.5
Indian	249306	21	6.9	4.2–11.2
Other	783019	212	21.8	17.2–27.2
**Education level**				
Primary or lower	1045699	245	28.7	24.2–33.6
Secondary	1990159	349	55.3	50.5–60.0
Tertiary	575035	92	16.0	12.1–20.8
**Marital status**				
Single	1151741	219	32.0	27.0–37.5
Married	2448152	467	68.0	62.5–73.0
**Age of smoking initiation** (years)				
<18	1639480	313	46.3	40.0–52.6
≥18	1903407	364	53.7	47.4–60.0
**Quit attempt in past 12 months**				
Yes	1615460	316	44.9	39.2–50.7
No	1935834	368	53.8	47.7–59.8
**Quit intention in next 12 months**				
Yes	691342	127	19.5	14.7–25.5
No	2849395	555	80.5	74.5–85.3
**Perceived difficulty in quitting**				
Not difficult	1093786	196	33.6	27.4–40.5
Difficult	2158689	430	66.4	59.5–72.6
**Apprehension about adverse health outcomes**				
Not worried	1434761	258	43.9	37.8–50.1
Worried	1835793	376	56.1	49.9–62.2

* Estimates are weighted to account for the complex survey design and non-response (total estimated population: 3599894, year 2020).

### Univariate associations with TTFC

In univariate analysis, TTFC within 30 minutes of waking was more prevalent among the Chinese ethnic (45.5%; 95% CI: 29.9–68.1), those initiated smoking at an age below 18 years (33.0%; 95% CI: 25.9–40.9), and smokers without a quit attempt in the past 12 months (36.3%; 95% CI: 29.8–43.4). Furthermore, current smokers who perceived difficulty in quitting (33.3%; 95% CI: 27.6–39.5) and respondents who were not worried at all about the adverse health impacts (39.6%; 95% CI: 32.1–47.5) were also significantly associated with TTFC within 30 minutes. However, there was no significant association between TTFC and other sociodemographic constituents like gender, education level, marital status, and quit intention (Table 2).

**Table 2:** Univariate analysis of TTFC among current smokers aged ≥15 years in Malaysia, by the sociodemographic and behavioral factors, Global Adult Tobacco Survey-Malaysia (GATS-M) 2023 (N=686)

*Variable*	*TTFC ≤30 minutes*			*TTFC >30 minutes*			*p*
	*Estimated population*	*Sample n*	*% (95% CI)*	*Estimated population*	*Sample n*	*% (95% CI)*	
**Gender**							
Male	1018227	222	29.2 (24.6–34.2)	2469137	445	70.8 (65.8–75.4)	0.93
Female	31311	5	27.8 (7.6–44.4)	81217	14	72.2 (35.6–92.4)	
**Ethnicity**							
Malay	628890	117	28.8 (23.3–35.1)	1554288	282	71.2 (64.9–76.7)	**0.002**
Chinese	174963	21	45.5 (29.9–68.1)	209426	33	54.5 (37.9–70.1)	
Indian	9314	3	3.7 (1.2–14.7)	239992	18	96.3 (85.3–99.1)	
Other	236370	86	30.2 (22.2–39.6)	546648	126	69.8 (60.4–77.8)	
**Education level**							
Primary or lower	355760	95	34.3 (26.3–43.4)	678939	150	65.5 (56.5–73.7)	0.28
Secondary	563535	109	28.3 (21.9–35.8)	1426623	240	71.7 (64.2–78.7)	
Tertiary	130243	23	22.6 (14.0–34.5)	444792	69	77.4 (65.5–86.0)	
**Marital status**							
Single	332610	74	28.9 (20.4–39.2)	819131	144	71.1 (60.8–79.6)	0.95
Married	716927	152	29.3 (23.6–35.7)	1731224	315	70.7 (64.3–76.4)	
**Age of smoking initiation** (years)							
<18	540591	116	33.0 (25.9–40.9)	1098889	197	67.0 (59.1–74.1)	**0.048**
≥18	508947	111	26.7 (20.4–34.2)	1394460	253	73.3 (65.8–79.6)	
**Quit attempt in past 12 months**							
Yes	325696	88	20.2 (15.3–26.1)	1289764	228	79.8 (73.9–94.7)	**0.036**
No	703485	138	36.3 (29.8–43.4)	1232348	230	63.7 (56.6–70.2)	
**Quit intention in next 12 months**							
Yes	139183	36	20.1 (12.1–31.5)	552159	91	79.9 (68.5–87.9)	0.080
No	879441	188	30.9 (26.0–36.2)	1969953	367	69.1 (63.8–74.0)	
**Perceived difficulty of quitting**							
Easy	212190	47	19.4 (12.9–28.2)	881596	149	80.6 (71.8–87.1)	**0.010**
Difficult	718327	166	33.3 (27.6–39.5)	1440362	264	66.7 (60.5–72.4)	
**Apprehension about adverse health outcome**							
Not worried	567760	107	39.6 (32.1–47.5)	867001	151	60.4 (52.5–67.9)	**<0.001**
Worried	401789	109	21.9 (16.7–28.1)	1434004	267	78.1 (71.9–83.3)	

TTFC: time to first cigarette upon waking up.

### Multivariable logistic regression analysis

After all variables significantly associated with the outcome from the univariate analysis have been adjusted in the multivariable model (Table 3), those with age of smoking initiation less than 18 years (AOR=1.60; 95% CI: 1.09–2.76) and those without a quit attempt in past 12 months (AOR=1.59; 95% CI: 1.39–2.01) were more likely to take the first cigarette within 30 minutes of waking. On the other hand, current smoking respondents with poor perception of quitting have also been identified as a significant determinant (AOR=1.48; 95% CI: 1.08–2.54). Those who reported not being worried about the adverse health outcomes of smoking were 2 times more likely to have a TTFC within 30 minutes, compared to their counterpart who had more concerns about the negative health impacts (AOR=2.04; 95% CI: 1.17–3.54).

**Table 3:** Multivariable analysis of associated factors of TTFC within 30 minutes of waking, among current smokers aged ≥15 years in Malaysia, Global Adult Tobacco Survey-Malaysia (GATS-M) 2023 (N=686)

*Variable*	*AOR*	*95% CI*	*χ^2^*	*p*
**Ethnicity**				
Malay	0.83	0.46–1.52	1.23	0.094
Chinese	1.37	0.54–3.45		
Indian	0.07	0.01–0.71		
Other (ref.)	1			
**Age of smoking initiation** (years)				
<18	1.60	1.09–2.76	5.84	**0.033**
≥18 (ref.)	1			
**Quit attempt in past 12 months**				
Yes (ref.)	1			
No	1.59	1.39–2.01	4.37	**0.036**
**Quit intention in next 12 months**				
Yes (ref.)	1			
No	1.40	0.69–2.87	0.77	0.38
**Perceived difficulty of quitting**				
Not difficult (ref.)	1			
Difficult	1.48	1.08–2.54	1.99	**0.042**
**Apprehension about adverse health outcomes**				
Not worried	2.04	1.17–3.54	6.25	0.012
Worried (ref.)	1			

AOR: adjusted odds ratio. TTFC: time to first cigarette.

All tested two-way interactions among the variables in the final model were non-significant and are therefore not presented. This indicates that the associations between each variable and TTFC were not significantly modified by any other variable in the model.

## DISCUSSION

This is the first local study to evaluate nicotine dependence based on the recognized item of TTFC, among the current smoking adult population in Malaysia, employing the most recent nationally representative sample. The measure of TTFC is factored as a core item in the FTND, and Heaviness of Smoking Index (HSI), which are the widely used global instruments^9^. The binary outcomes were investigated in the present study (TTFC within 30 minutes or more than 30 minutes), given that smoking the first cigarette within 30 minutes of waking up has been widely considered by the literature as a strong indicator of nicotine dependence^9^. Among existing smokers in Malaysia, the prevalence of TTFC within 30 minutes of waking was 29.2%. Among all items of FTND, TTFC demonstrated the strongest predictive validity related to cessation outcomes^8,9^. The TTFC prevalence, hence, indicates smoking dependence, in which regions or populations with a higher proportion of smokers reporting TTFC within 30 minutes typically have a more severely nicotine-dependent smoking population^15^. Our prevalence was lower compared to United States (59.5%)^16^ and Korea (53.9%)^17^, but higher than China (12.8%)^12^ though the Chinese study did not represent the entire adult smoking population.

The discrepancies in prevalence of TTFC within 30 minutes might be due to the variations in sociocultural-norms and even smoking behavior across nations, as well as the disparities in the survey methodology. In our study, the GATS specifically recruited adults aged ≥15 years as a compliance to the standardized component of the WHO’s Global Tobacco Surveillance System (GTSS). Conversely, the Korean study focused exclusively on middle-aged male current smokers^17^, while the Chinese study targeted the elderly population^12^ rendering the global burden of TTFC difficult to compare directly. While our analysis focuses specifically on the 2023 GATS Malaysia dataset, the 2011 GATS Malaysia Fact Sheet provides a valuable historical baseline for comparison. The 2011 report indicated that almost half of all current daily smokers had their first cigarette within 30 minutes of waking^18^. By contrast, our current 2023 analysis shows that this proportion has been reduced to less than one-third. This comparison suggests a significant downward trend in nicotine dependence levels over the past 12 years, likely reflecting the positive impact of cumulative cessation support and tobacco control policies in Malaysia. Our finding also indicated a favorable pattern of increases in quit attempts. The percentage of Malaysian smokers who reported quitting smoking grew from 24.0% in 2012 to 50.6% in 2020^19^. Additionally, the number of smokers registered under the mQuit program, which was developed under the National Strategic Plan on Tobacco Control in order to strengthen cessation services in Malaysia, had drastically increased from 7757 (2015) to 28167 (2020)^20^. These longitudinal observations, when paired with our current 2023 findings, underscore the importance of sustained cessation services in reducing high-dependence smoking patterns.

Past literature has identified individual sociodemographic, economic and occupational factors as the primary determinants of nicotine dependence^21^. The present study highlights smoking behavioral cues that significantly determined TTFC upon waking among the current smokers nationwide, including age of smoking initiation, quit attempt in the past, perception of quitting difficulty, and apprehension about negative health impacts of smoking.

The odds of consuming the first cigarette within 30 minutes of waking up were 1.6 times significantly higher among those who initiated smoking aged <18 years. The underlying mechanism for this heightened dependence among early initiators is largely attributed to the developing brain during adolescence. Adolescents’ brains are still developing significantly, especially in areas linked to decision-making, reward, and impulse control. Therefore, nicotine exposure during this critical period can lead to profound and rapid neuroadaptations in the brain’s reward pathways, specifically by influencing the dopamine and nicotinic acetylcholine receptors (nAChRs), increasing the susceptibility to addiction and decreasing the ability to control cravings^22^. Consequently, the earlier exposure to tobacco products predisposes the brain for stronger dependence, implying that the withdrawal symptoms of intense cravings upon waking after a period of abstinence due to an overnight sleep, become more severe and immediate^22^. Studies in Japan^23^ and the European Union^24^ consistently show that early smoking initiation is associated with higher FTND scores and lower cessation rates. These scenarios indicate the universal nature of nicotine’s impact on the developing brain, regardless of geographical location. The substantial association between early initiation and severe dependence contributes to a significant public health burden. Consequently, delaying smoking initiation through a combined public health intervention, such as an impactful school health educational program, and stricter enforcement of the relevant regulation on the legal age for tobacco purchase, are among the crucial measures to reduce overall nicotine dependence and combat smoking habits during pre-adulthood.

In the present study, current smokers without a previous quit attempt in the past 12 months were significantly more likely to report TTFC within 30 minutes of waking, a commonly used indicator of higher nicotine dependence. Nevertheless, this significant pattern of association was not observed in quit intention in the next 12 months. The relationship between nicotine dependence, quit attempts, and quit intentions is complex and bidirectional. While the absence of recent quit attempts in our analysis was associated with earlier TTFC, this does not imply a simple linear pathway whereby stronger dependence uniformly prevents quit attempts. Highly dependent smokers may make repeated but unsuccessful quit attempts, whereas others may refrain from attempting to quit due to low self-efficacy, anticipated withdrawal symptoms, or reduced perceived behavioral control. Conversely, repeated failed attempts may undermine confidence and reduce subsequent motivation to quit, as described in the Theory of Planned Behavior^25^.

While both past quit attempts and future quit intentions are relevant, they represent conceptually distinct constructs: past quit attempts reflect enacted behavior, whereas quit intentions reflect motivational readiness that may or may not translate into action. However, intentions to quit in the future, while also linked to dependence, may be influenced by ambivalence, social desirability, and perceived behavioral control, and therefore do not consistently predict an actual quit attempt^26^. Within the constraints of the WHO standardized GATS instrument, aligned with MPOWER indicators, past 12-month quit attempts and intention to quit in the next 12 months are measured as dichotomous indicators. While these standardized measures enhance comparability across countries, they do not capture the frequency, duration, intensity, or context of quit attempts, nor the strength or immediacy of quit intentions. The concept that a lack of quit attempts is linked to increased nicotine addiction, reinforcing the addiction cycle and hindering self-efficacy for quitting, may partially explain the observed association in this population, although alternative pathways are plausible. This phenomenon is grounded in the understanding of operant conditioning and social cognitive theory. Repeated exposure to nicotine without interruption or challenge (quit attempts) provides continuous positive reinforcement for smoking behavior, strengthening the neural pathways associated with tobacco-seeking and tobacco-taking^27^.

This finding also highlights that intentions are an important intermediate step toward quitting, but do not guarantee a successful or even initiated quit attempt. Thus, behavioral interventions in cessation support play a crucial role in supporting smokers who remain ambivalent or have low self-perceived control, helping them move towards action. A meta-analysis on behavioral interventions for tobacco cessation in low- and middle-income countries has emphasized techniques like motivational interviewing help individuals explore their personal reasons for quitting, weigh the pros and cons, and develop a sense of confidence in their ability to succeed. By tailoring the intervention to the individual’s specific stage of change and their unique circumstances, these approaches help to crystallize a smoker’s intent to quit into a concrete plan, such as setting a quit date and preparing for potential challenges^28^. Such stage-matched and self-efficacy enhancing interventions may help bridge the gap between passive contemplation and active quit attempts, particularly among smokers with higher levels of dependence who face greater physiological and psychological barriers to cessation.

The present study revealed that smokers with a self-perceived difficulty in quitting was a significant psychological factor of nicotine dependence. This association can be understood via several interconnected psychological and physiological mechanisms. The self-perceived difficulty in quitting is a measure of self-efficacy, which indicates the smokers’ belief in their own ability to succeed at quitting^29^. Low self-efficacy in quitting smoking is a powerful predictor of relapse and is often a reflection of past failed attempts, which can lead to a sense of hopelessness and a perception that the addiction is insurmountable^28,30^. The psychological aspect of self-perceived difficulty in quitting, or barriers, is directly intertwined with this physiological drive. Smokers who feel they have little control over their addiction are more likely to succumb to the intense morning cravings. This behavior, in turn, reinforces their belief that quitting is difficult or impossible, creating a vicious cycle^9^. The act of reaching for a cigarette within 30 minutes of waking is not only a habit, but more likely a rapid response to a powerful physiological craving, which is fueled by a psychological state of perceived helplessness^31^. Therefore, a shorter TTFC is a behavioral manifestation of both high physiological dependence and the psychological conviction that quitting is an impossible task^15^. Additionally, the Theory of Planned Behavior (TPB) posits that an individual’s perception may influence specific behavioral participation. The TPB proposes that a person’s desire to engage in a behavior will be stronger if they have a more positive attitude, a more favorable subjective norm or perception, and a larger sense of behavioral control^25^. This theoretical framework provides a robust explanation for the link between cognitive beliefs and actual actions across a wide range of domains, especially in health behaviors. Therefore, recognizing self-perceived difficulty in quitting as an important determinant, cessation interventions should concentrate on an educational approach in par with boosting smokers’ confidence in their capacity to quit in order to increase their self-efficacy. Depending on the person’s stage of transformation, special techniques including group support groups, cognitive behavioral therapy (CBT), and motivational interviewing have been proven as effective tools^32^.

In our study, individuals who did not have an apprehension about the adverse health outcomes were two times significantly more likely to smoke the first cigarette within 30 minutes of waking up. Similar findings were found in an Indian study that nicotine dependence and health risk apprehension are intertwined, with higher levels of nicotine dependence often linked to lower awareness of health risks associated with smoking, especially the extra cardiopulmonary disease such as stroke and reproductive health effects (infertility, low birth weight, genital cancer)^33^. The phenomenon is best explained by numerous social-cognitive theories of health behavior, especially the Health Belief Model and Protection Motivation Theory which propose that deliberative and reflective cognitions motivate action^34^. One cognition associated with risk is worry, which is an anticipatory negative emotion experienced when thinking about possible future events. Worry about health consequences has been reported to be associated with motivation or intention to quit smoking, in a population study in England^35^, which explains individuals who downplay the health risks associated with smoking may exhibit higher levels of nicotine dependence. Therefore, targeting the behavioral mechanism that drives nicotine dependence is crucial for the application of the behavioral modification approach in the comprehensive nicotine cessation support among existing smokers.

The behavioral component plays a major role in determining nicotine dependence, which predicts the likelihood of successful quitting. From a public health perspective, cessation strategies should consider individual behavioral triggers and perceptions identified in empirical findings. While comprehensive tobacco control policies aligned with the WHO FCTC framework, including tobacco taxation, restricting points of sale, enforcing advertising bans, and reducing environmental smoking cues, are fundamental in reducing tobacco use at the population level, individual-level behavioral mechanisms should not be overlooked, particularly among current smokers attempting cessation. Structural interventions reduce exposure and normalize cessation; however, highly dependent smokers may still require targeted behavioral support to address cue-induced cravings and perceived difficulty in quitting. By understanding the specific cognitive and behavioral processes associated with higher dependence, public health initiatives can complement policy measures with targeted, evidence-based behavioral interventions^36^. Public health programs that educate individuals to recognize and manage cue-induced cravings through cognitive-behavioral techniques delivered via quit lines, digital applications, or community health interventions, are essential for achieving sustained and long-term abstinence^35^, especially when integrated within supportive regulatory environments.

### Limitations

This study has several limitations. First, the cross-sectional design restricts the examination of the causal effect relationship between TTFC and the sociodemographic behavioral determinants. Additionally, the self-reported data collection may be subject to recall bias and reporting bias, including social desirability bias, as tobacco-related behaviors may be underreported due to local social norms that discourage such practices. Although we adjusted for key confounders using multivariable logistic regression, residual confounding due to unmeasured or imperfectly measured variables cannot be entirely excluded. Furthermore, behavioral determinants in the questionnaire adapted were only assessed by a single-item measure, which is convenient for brevity but limits the psychometric standpoint. In addition, the study sample of current smokers was overwhelmingly male, with a very small proportion of female smokers included in the analysis. Although this distribution reflects national smoking patterns within the Malaysian sociocultural context, findings related to gender differences in TTFC, including the absence of significant associations, should be interpreted with caution due to the limited number of female respondents and the relatively small sample sizes in several subgroup analyses. Nonetheless, the study employed a nationwide multistage stratified sampling design, enhancing representativeness and enabling nationally generalizable estimates for the adult Malaysian population. Data collection adhered to a globally standardized WHO protocol, and all research assistants received standardized training to ensure consistency and data quality.

## CONCLUSIONS

This study revealed that less than one-third of Malaysian current smokers reported consuming their first cigarette within 30 minutes of waking. Subpopulations with specific behavioral cues, including those who initiated smoking before the age of 18 years, smokers without previous quit attempt, those who perceived quitting as difficult, and those who are not worried about the adverse health outcomes, were significantly associated with TTFC. This finding implies that nicotine dependence among individual smokers with lower self-efficacy must be critically addressed through impactful behavior-based interventions. Although comprehensive tobacco control efforts should address the entire population, the identified high-risk smoking subgroups may benefit from more focused and tailored behavioral interventions. These findings provide important evidence to inform the refinement of anti-tobacco initiatives targeting smokers with indicators of stronger nicotine dependence.

## Data Availability

The dataset has been disseminated to the WHO GATS data system.
